# Large-scale genomics unveil polygenic architecture of human cortical surface area

**DOI:** 10.1038/ncomms8549

**Published:** 2015-07-20

**Authors:** Chi-Hua Chen, Qian Peng, Andrew J. Schork, Min-Tzu Lo, Chun-Chieh Fan, Yunpeng Wang, Rahul S. Desikan, Francesco Bettella, Donald J. Hagler, Pediatric Imaging, Neurocognition and Genetics Study, Connor McCabe, Linda Chang, Natacha Akshoomoff, Erik Newman, Thomas Ernst, Peter Van Zijl, Joshua Kuperman, Sarah Murray, Cinnamon Bloss, Mark Appelbaum, Anthony Gamst, Wesley Thompson, Hauke Bartsch, Alzheimer's Disease Neuroimaging Initiative, Michael Weiner, Paul Aisen, Ronald Petersen, Clifford R. Jack Jr, William Jagust, John Q. Trojanowki, Arthur W. Toga, Laurel Beckett, Robert C. Green, Andrew J. Saykin, John Morris, Leslie M. Shaw, Zaven Khachaturian, Greg Sorensen, Maria Carrillo, Lew Kuller, Marc Raichle, Steven Paul, Peter Davies, Howard Fillit, Franz Hefti, Davie Holtzman, M. Marcel Mesulman, William Potter, Peter J. Snyder, Adam Schwartz, Tom Montine, Ronald G. Thomas, Michael Donohue, Sarah Walter, Devon Gessert, Tamie Sather, Gus Jiminez, Danielle Harvey, Matthew Bernstein, Nick Fox, Paul Thompson, Norbert Schuff, Charles DeCarli, Bret Borowski, Jeff Gunter, Matt Senjem, Prashanthi Vemuri, David Jones, Kejal Kantarci, Chad Ward, Robert A. Koeppe, Norm Foster, Eric M. Reiman, Kewei Chen, Chet Mathis, Susan Landau, Nigel J. Cairns, Erin Householder, Lisa Taylor-Reinwald, Virginia M.Y. Lee, Magdalena Korecka, Michal Figurski, Karen Crawford, Scott Neu, Tatiana M. Foroud, Steven Potkin, Li Shen, Kelley Faber, Sungeun Kim, Kwangsik Nho, Leon Thal, Richard Frank, Neil Buckholtz, Marilyn Albert, John Hsiao, Lars T. Westlye, William S. Kremen, Terry L. Jernigan, Stephanie Le Hellard, Vidar M. Steen, Thomas Espeseth, Matt Huentelman, Asta K. Håberg, Ingrid Agartz, Srdjan Djurovic, Ole A. Andreassen, Nicholas Schork, Anders M. Dale

**Affiliations:** 1 Multimodal Imaging Laboratory, Department of Radiology, University of California San Diego, La Jolla, California 92037, USA.; 2 Department of Human Biology, J. Craig Venter Institute, San Diego, California 92037, USA.; 3 Department of Molecular and Cellular Neuroscience, The Scripps Research Institute, La Jolla, California 92037, USA.; 4 Department of Cognitive Science, University of California, San Diego, La Jolla, California 92093, USA.; 5 Department of Neurosciences, University of California, San Diego, La Jolla, California 92093, USA.; 6 Norwegian Center for Mental Disorders Research (NORMENT), KG Jebsen Centre for Psychosis Research, Institute of Clinical Medicine, University of Oslo, 0424 Oslo, Norway.; 7 NORMENT, KG Jebsen Centre for Psychosis Research, Department of Psychology, University of Oslo, 0424 Oslo, Norway.; 8 NORMENT, KG Jebsen Centre for Psychosis Research, Division of Mental Health and Addiction, Oslo University Hospital, 0317 Oslo, Norway.; 9 Department of Psychiatry, University of California, San Diego, La Jolla, California 92093, USA.; 10 VA San Diego Center of Excellence for Stress and Mental Health, La Jolla, California 92037, USA.; 11 Dr E. Martens Research Group of Biological Psychiatry, Center for Medical Genetics and Molecular Medicine, Haukeland University Hospital, 5021 Bergen, Norway.; 12 NORMENT, KG Jebsen Centre for Psychosis Research, Department of Clinical Science, University of Bergen, 5021 Norway.; 13 Translational Genomics Research Institute, Phoenix, Arizona 85004, USA.; 14 Department of Neuroscience, Norwegian University of Science and Technology (NTNU), 7489 Trondheim, Norway.; 15 Department of Medical Imaging, St. Olav's University Hospital, 7006 Trondheim, Norway.; 16 Department of Psychiatric Research, Diakonhjemmet Hospital, 0319 Oslo, Norway.; 17 Department of Medical Genetics, Oslo University Hospital, 0424 Oslo, Norway.; 18 UC San Diego, La Jolla, CA 92093, USA.; 19 U Hawaii, Honolulu, HI 96822, USA.; 20 Kennedy Krieger Institute, Baltimore, MD 21205, USA.; 21 Scripps Translational Science Institute, La Jolla, CA 92037, USA.; 22 UC San Francisco, San Francisco, CA 94143, USA.; 23 Mayo Clinic, Rochester, MN 55905, USA.; 24 UC Berkeley, Berkeley, CA 94720-5800, USA.; 25 U Pennsylvania, Philadelphia, PA 19104, USA.; 26 USC, University of Southern California, Los Angeles, CA 90033, USA.; 27 UC Davis, Davis, CA 95616, USA.; 28 Brigham and Women s Hospital/Harvard Medical School, Boston MA 02115, USA.; 29 Indiana University, Indianapolis, IN 46202-5143, USA.; 30 Washington University St. Louis, St. Louis, MO 63130, USA.; 31 Prevent Alzheimer's Disease 2020, Rockville, MD 20850,USA.; 32 Siemens; 33 Alzheimer's Association, Chicago, IL 60601, USA.; 34 University of Pittsburgh, Pittsburgh, PA 15260, USA.; 35 Cornell University, Ithaca, NY 14850, USA.; 36 Albert Einstein College of Medicine of Yeshiva University, Bronx, NY 10461, USA.; 37 AD Drug Discovery Foundation, New York, NY 10019, USA.; 38 Acumen Pharmaceuticals, Livermore, California 94551, USA.; 39 Northwestern University, Evanston, IL 60208, USA.; 40 National Institute of Mental Health, Bethesda, MD 20892-9663, USA.; 41 Brown University, Providence, RI 02912, USA.; 42 Eli Lilly, Indianapolis, Indiana 46285, USA.; 43 University of Washington, Seattle,WA 98195, USA.; 44 University of London, LondonWC1E 7HU, UK.; 45 University of Michigan, Ann Arbor, MI 48109, USA.; 46 University of Utah, Salt Lake City, UT 84112, USA.; 47 Banner Alzheimer's Institute, Phoenix, AZ 85006, USA.; 48 UC Irvine, Irvine, CA 92697, USA.; 49 General Electric; 50 National Institute on Aging/National Institutes of Health, Bethesda, MD 20892, USA.; 51 The Johns Hopkins University, Baltimore, MD 21218, USA.

## Abstract

Little is known about how genetic variation contributes to neuroanatomical variability, and whether particular genomic regions comprising genes or evolutionarily conserved elements are enriched for effects that influence brain morphology. Here, we examine brain imaging and single-nucleotide polymorphisms (SNPs) data from ∼2,700 individuals. We show that a substantial proportion of variation in cortical surface area is explained by additive effects of SNPs dispersed throughout the genome, with a larger heritable effect for visual and auditory sensory and insular cortices (*h*^2^∼0.45). Genome-wide SNPs collectively account for, on average, about half of twin heritability across cortical regions (*N*=466 twins). We find enriched genetic effects in or near genes. We also observe that SNPs in evolutionarily more conserved regions contributed significantly to the heritability of cortical surface area, particularly, for medial and temporal cortical regions. SNPs in less conserved regions contributed more to occipital and dorsolateral prefrontal cortices.

## Abstract (web summary)

How genetic variation contributes to brain morphology is still poorly understood. Here Chen *et al*. combine brain imaging with single-nucleotide polymorphism data to discover that a substantial degree of cortical variation is derived from underlying genetic differences.

## Graphical abstract

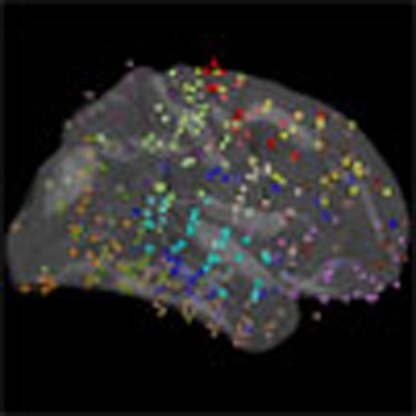

To understand the complexity of human higher cognition, it is essential to study the properties of the cerebral cortex^1^,^2^. Genetics play a critical role, as identifying the genetic underpinning of phenotypic variability provides a causal foothold. A striking feature of the human cerebral cortex is that it follows an ancient mammalian prototype but also displays an enormous expansion in cortical surface area^3^,^4^,^5^,^6^. This expansion did not occur homogeneously across the cortex^1^,^3^. The alteration of cortical organization may have significant functional consequences in human cognition. Genetic variation is thought to be a major factor in this alteration and to underlie phenotypic variability among individuals^1^. However, quantifying the source of genetic contribution to phenotypic differences in humans and mapping the genetic and evolutionary architecture of cortical surface area across different cortical regions are ongoing challenges.

One fundamental question is the extent to which neuroanatomical variability among individuals is caused by genetic differences. Twin and pedigree designs have been used to estimate the ‘heritability' of a phenotype by examining the resemblance of the phenotype between relatives^7^. Heritability is the proportion of the phenotypic variance ascribable to genetic differences in a given population: the proportion of variation due to additive genetic effects (narrow-sense heritability) or the proportion of variation due to all genetic effects (broad-sense heritability)^7^. Twin/family studies have shown that brain phenotypes are heritable (for example, heritability up to ∼0.8)^8^.

Technological advances now allow assay of individuals for millions of single-nucleotide polymorphisms (SNPs) spanning the whole genome^9^. Genetic similarity or relationship among a group of individuals can then be estimated through the use of dense genetic variants. By contrasting genetic similarity with phenotypic similarity, one can estimate the heritability of a phenotype in the absence of family members^9^,^10^. A recent popular method for carrying out relevant analyses involves a mixed linear model to fit a genetic relationship matrix (GRM) to measured phenotypes, such as the methodology built into the genome-wide complex trait analysis (GCTA) tool^9^,^11^. The resulting estimate is referred to as ‘SNP' or ‘chip heritability' (*h*^2^)^12^,^13^. Using the GCTA approach, researchers have estimated that about half of the heritability of human height can be attributed to ∼0.3 million common SNPs^11^. In comparison, only ∼16% of variability in height can be attributed to all individual SNPs discovered by genome-wide association studies^14^. This finding suggests that height has a polygenic architecture in which a large number of common genetic variants with small effects contribute predominantly additively to phenotypic variation.

To investigate the polygenic contribution of common SNPs to cortical structures, we apply the GCTA method to a combined sample from five cohorts. Raw imaging and genotype data from all study cohorts are processed with a standardized protocol to minimize data heterogeneity. The analysis for estimating SNP heritability is potentially sensitive to population structure (that is, population stratification and cryptic relatedness). It is therefore typical to restrict the analysis to unrelated individuals of a single genetic ancestry^12^. We exclude non-Europeans based on principal component analysis of the GRM. We estimate pairwise GRM using all 2,480,482 directly genotyped and imputed autosomal SNPs. We also exclude related individuals using two thresholds at an estimated GRM ≥0.025 (more related than third or fourth cousins), or a less stringent threshold at an estimated GRM ≥0.1. This sampling results in subsets of 2,364 or 2,698 generally unrelated individuals with European ancestry, respectively.

Phenotype definition is critical for all genetic association studies, especially in brain imaging genetic studies, due to the high dimensionality of cortical measures (∼0.3 million points per subject)^15^. Using a data-driven, fuzzy clustering technique with magnetic resonance imaging (MRI) scans of twins, we previously parcelled cortical surface area into 12 genetic subdivisions, creating an atlas based solely on genetically informative data^6^,^16^ (Fig. 1b). Boundaries of the genetic divisions correspond largely to meaningful structural and functional regions; however, the divisions represent novel phenotypes. We use these regions, conforming to the genetic patterning of cortical surface area, to increase power for detecting effects and to minimize multiple comparisons after reducing image dimensionality to these parcels. The aim of our large-scale whole-genome and whole-cortex analyses is to examine and dissect the polygenic genetic architecture of cortical surface area across different cortical regions.

## Results

### SNP heritability

**Figure 1:**
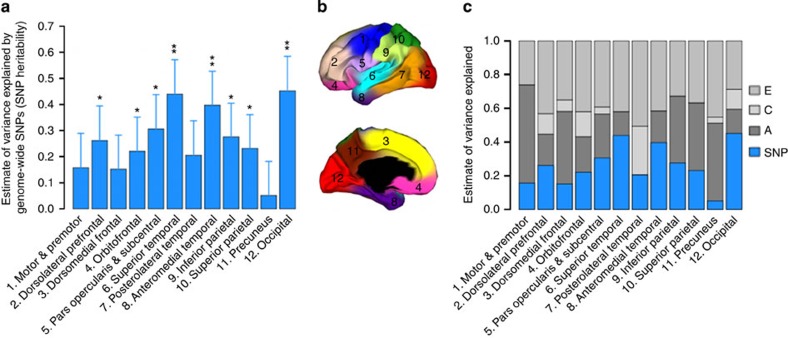
SNP heritability. (**a**) Estimates of variance explained by all autosomal SNPs for each cortical region (SNP heritability) from genetic relationships <0.025 (GRM<0.025). Error bars represent the s.e. of the estimates. Estimates were tested for significantly different from zero by likelihood ratio test comparing the full and reduced models. *P<0.05, **P<0.004 (Bonferroni correction threshold). (**b**) Genetic clustering map shows the anatomical location of the cortical phenotypes^16^. (**c**) SNP heritability estimates (blue bars) are overlaid on twin heritability estimates (grey bars). Shared environmental variances are small and not significant for all phenotypes. A: additive genetic variance; C: shared environmental variance; E: unique environmental variance.

In our five-cohort sample, we found that a substantial proportion of variation in surface area in almost all of the 12 regions is captured by all autosomal SNPs after accounting for global cortical size. A few cortical regions, such as the insula, visual and auditory sensory regions, including superior temporal and occipital cortices, have a high SNP heritability of up to ∼0.45 (s.e. 0.12) (Fig. 1a; Supplementary Table 1). We reported two sets of results based on the inclusion of individuals with pairwise GRM entry scores <0.025 or <0.1 to determine the consistency of our findings. The GRM<0.1-sample offers the advantage of a larger sample size, whereas the GRM<0.025-sample is less susceptible to potential confounding from cryptic relatedness. The results from the two sets of samples are consistent (Supplementary Fig. 1a; Supplementary Table 1), supporting the reliability of our findings. To ensure the validity of the method applied to our sample, we performed a simulation study and power calculation. We also verified that our main findings were not sensitive to patient samples, nor sensitive to linkage disequilibrium (LD), that is, the correlation among SNPs, in the genome (see Supplementary Methods and Supplementary Table 2).

### Twin heritability

We next sought to compare the SNP heritability with twin heritability estimated from 466 twins. Heritabilities of the same cortical imaging phenotypes were estimated in a classical twin model implemented in the OpenMx software suite. The results of twin heritability estimates (additive genetic variances) across different cortical regions are shown in Fig. 1c (Supplementary Table 3)^8^. The average monozygotic and dizygotic correlations across all cortical regions were 0.62 and 0.32, respectively, suggesting almost a perfect additive genetic proportion in these phenotypes.

### Partitioning of genomic variation by genic annotation

**Figure 2:**
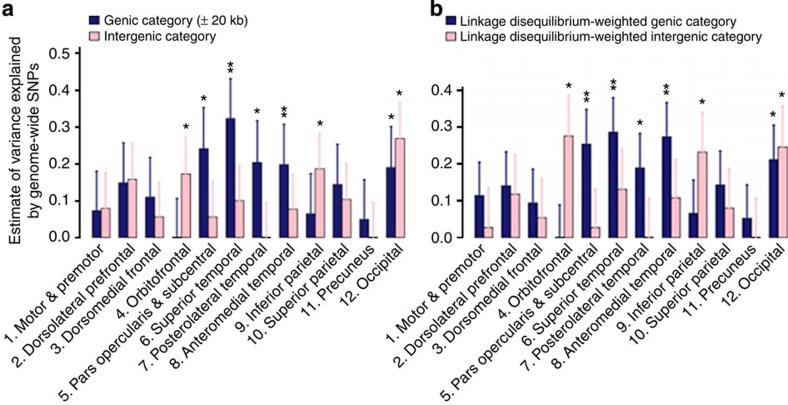
Partitioning of genomic variation by genic annotation. (**a**) Estimates of variance explained by genic and intergenic regions (GRM<0.025). The genic region is defined as ±20 kb from the 3′ and 5′ UTRs. (**b**) Estimates of variance explained by genic and intergenic regions. The genic region is defined by the LD-weighted genic annotation scheme. *P<0.05, **P<0.004. Error bars represent the s.e. of the estimates. Estimates were tested for significantly different from zero by likelihood ratio test.

We partitioned the variance explained by all the SNPs into genic and intergenic regions across all autosomal chromosomes. We defined genic boundaries as 20 kb upstream and downstream from the 3′ and 5′ untranslated regions (UTRs) of each gene^17^ (Fig. 2a; Supplementary Tables 4 and 7). Furthermore, we used an LD-weighted genic annotation scheme that takes into account the LD structure to categorize SNPs that have high LD with SNPs within genic elements (Fig. 2b; Supplementary Tables 5 and 8)^18^. The results from the two methods were consistent, with evidence of enriched genetic effects in the genic regions for many cortical regions.

### Partitioning of genomic variation by conservation annotation

**Figure 3:**
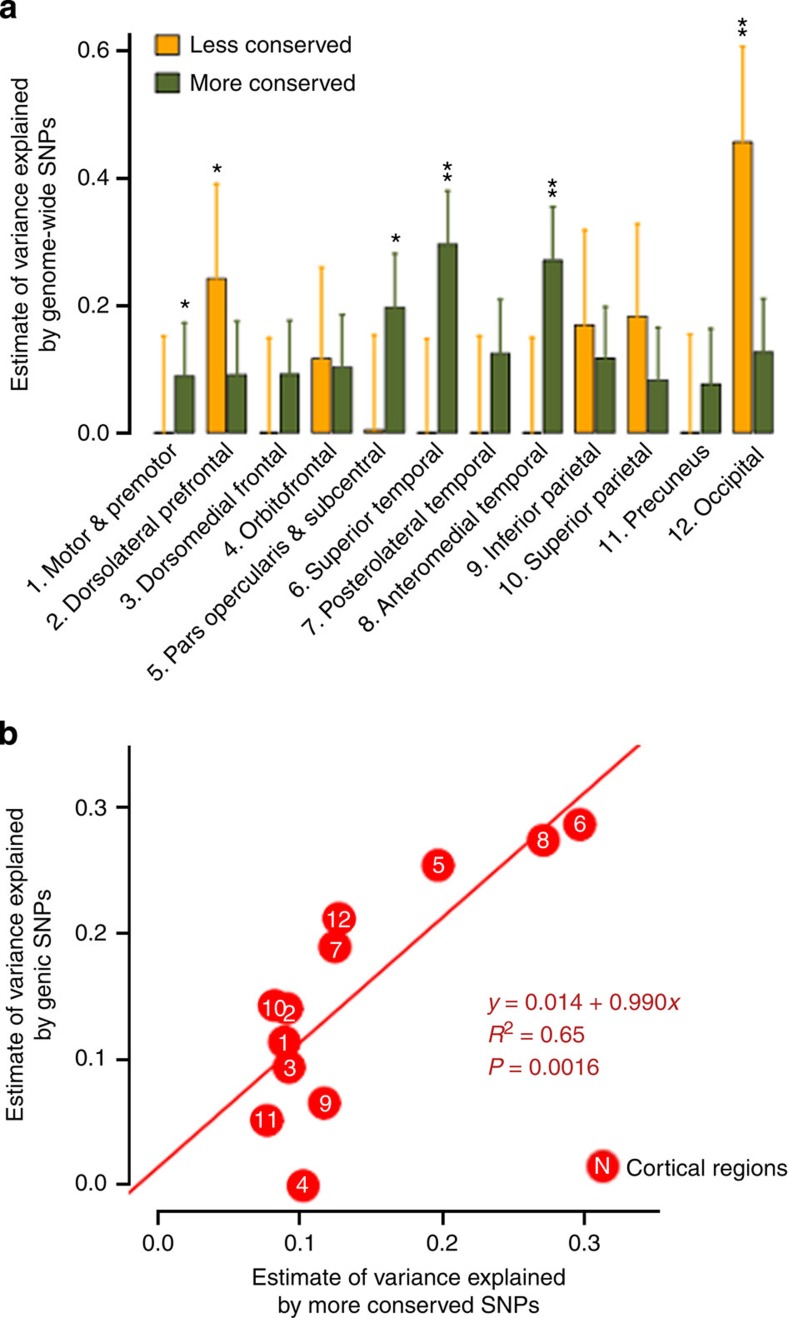
Partitioning of genomic variation by conservation annotation. (**a**) Estimates of variance explained by less conserved and more conserved regions (GRM<0.025). *P<0.05, **P<0.004. Error bars represent the s.e. of the estimates. Estimates were tested for significantly different from zero by likelihood ratio test. (**b**) A significant correlation between estimates of variance explained by genic and more conserved SNPs across phenotypes.

Conservation scores were derived from multiple alignments of placental mammal genomes to the human genomes^19^. We observed that more conserved SNPs collectively have genetic influences on several cortical regions especially around the insula, superior, anterior and medial temporal lobes, including parahippocampus gyrus and entorhinal cortex (Fig. 3a; Supplementary Table 6). Less conserved SNPs collectively exhibit greater polygenic effects on occipital and dorsolateral prefrontal cortices.

### Correlation explained by genic and conserved SNPs

We also found a highly significant correlation between the variance explained by genic SNPs and variance explained by more conserved SNPs (Fig. 3b), and between intergenic SNPs and less conserved SNPs (Supplementary Fig. 3). To rule out the possibility that genic and conservation annotation are surrogates for one another, we computed the correlation between the LD-weighted genic and conservation scores. The correlation of *r*=0.58 indicates that substantial variation can be attributed uniquely to each (shared variance, *R*^2^=0.34).

## Discussion

Although identifying genetic determinants of the human brain is an active area of research^20^,^21^, studies on the polygenic architecture of brain imaging phenotypes are limited, partially because of a lack of availability of appropriate data sets^22^,^23^. We show that a substantial proportion of the heritable component of the cortex resides among common variants that can be interrogated via current genome-wide genotyping arrays. This suggests that with larger sample sizes, SNPs associated with cortical surface area could be discovered^24^. A few cortical regions, such as the insula, visual and auditory sensory regions, including superior temporal and occipital cortices, have a high SNP heritability of up to ∼0.45 (s.e. 0.12). Recent evidence suggests that non-heritable genetic variation might be widespread in the brain and has potential contribution to complex functional diversification^25^,^26^. Our results show that heritable genetic variation has substantial impact on cortical area variation. Thus, these findings imply that genetic underpinnings of brain phenotypes likely involve the combined effects of many common variants of small effects, as well as non-heritable genetic variation.

SNP heritability estimates quantify the overall contribution of the additive effects of all SNPs, which provides a lower bound of the narrow-sense heritability of the trait estimated in pedigree studies, since pedigree information captures the effects of all genetic variants on phenotypic similarity^13^. We observe broad agreement between SNP and twin heritability across cortical regions. Similar to the height study^11^, we captured about half of twin heritability on average with the SNP heritability across cortical regions. However, some regions, such as the motor–premotor cortex and precuneus, have high twin heritability but low SNP heritability. This finding suggests that non-additive genetic effects could play a role in these phenotypes, because the additive genetic effects estimated from the twin model potentially include non-additive effects such as epistatic interactions and inherited epigenetic variation^27^,^28^. Alternatively, ungenotyped causal variants affecting these regions might have lower allele frequencies than do common SNPs, and/or are not tagged by the genotyped SNPs. Furthermore, the difference could also be due purely to sampling variation—including differences in age, gender and ancestry—or random errors (for example, both twin and SNP heritability estimates have average s.e. of ∼0.11). Taken together, the observed information provides clues, and lower and upper bounds of genetic effects, in the search for trait-associated variants.

Obtaining evidence for the polygenic architecture of complex traits provides a rationale for further dissecting the contribution of particular genomic regions to phenotypic expression^10^,^17^,^29^. Specifically, we focus on the genetic effects of genic and regulatory element regions of the genome. SNPs in these functional genomic regions have been shown to be enriched for associations across diverse phenotypes^18^. We therefore partitioned the variance explained by all the SNPs into genic and intergenic regions across all autosomal chromosomes. We defined genic boundaries as 20 kb upstream and downstream from the 3′ and 5′ UTRs of each gene^17^ (Fig. 2a; Supplementary Table 4). Furthermore, we used an LD-weighted genic annotation scheme that takes into account the LD structure to categorize SNPs that have high LD with SNPs within genic elements (Fig. 2b; Supplementary Table 5)^18^. The results from the two methods were consistent, with evidence of enriched genetic effects in the genic regions for many cortical regions. However, some cortical regions also had substantial variation explained by SNPs partitioned into an intergenic category, such as the occipital, orbitofrontal and inferior parietal cortices. Note that the intergenic category in this definition may still include some regulatory elements farther away from genes (for example, enhancers); these have been implicated in brain development^30^.

The 1000-fold difference in cortical surface area between humans and mice may contribute to our complex behaviours^1^,^3^. The cerebral cortex subserves an array of higher-order brain functions that are uniquely specialized in humans, and changes in these functions and their networks may make us prone to neurobiological disorders such as schizophrenia, autism or Alzheimer's disease^1^,^2^,^4^,^31^. Therefore, it is of particular interest to explore the contribution of genetic variants that are presumably more human specific in their evolutionary lineage than other variants^31^. Conservation scores were derived from multiple alignments of placental mammal genomes to the human genomes^19^. We observed that more conserved SNPs collectively have genetic influences on several cortical regions especially around the insula, superior, anterior and medial temporal lobes, including parahippocampus gyrus and entorhinal cortex (Fig. 3a; Supplementary Table 6). Several of these regions belong to the allocortex, which has fewer cortical laminae than the neocortex and is regarded as evolutionarily more primitive^32^. On the other hand, less conserved SNPs collectively exhibit greater polygenic effects on occipital and dorsolateral prefrontal cortices, the regions that subserve visual perception and executive function respectively. Visual specialization is one hallmark of primate brain evolution. Primates have relatively enlarged visual areas and are visually orientated mammals^33^. The dorsolateral prefrontal cortex is located in the expanded prefrontal cortex of primates, and is a vital region of distributed brain networks linked to many complex cognitive functions in humans^5^.

We also found a highly significant correlation between the variance explained by genic SNPs and variance explained by more conserved SNPs (Fig. 3b), and between intergenic SNPs and less conserved SNPs (Supplementary Fig. 3). These findings suggest the existence of a possible pattern in which phenotypic variation in more conserved cortical regions is influenced to a greater degree by more conserved and genic SNPs, and more human-specific cortical regions are influenced to a greater degree by less conserved and intergenic SNPs. Our result is preliminary, but this trend may be biologically plausible and is noteworthy for further investigation.

We show that a substantial proportion of the heritable component of the cortex resides among common variants that can be interrogated via current genome-wide genotyping arrays. By leveraging genic and conservation annotations we were able to reveal that particular genomic regions are enriched for variants that influence variation in cortical surface area. Each cortical region appears to have elements of region-specific genetic architecture, which might relate to functional specialization of the cortical regions. Elucidating the sources of these genetic effects will allow investigators to prioritize resources for future investigations. Cortical surface area is similar to other complex traits in terms of polygenicity distributed among common variants and genetic effects enriched in genic regions. Yet, the human brain is a uniquely complex phenotype, in that its genomic properties appear as complex as its functional capacity. In this light, it should be acknowledged that beyond polymorphisms and structural variants in the genome, epigenomics^34^, alternative splicing^35^ and somatic mosaicism^25^ may contribute to phenotypic diversity in normal brain development. A variety of data types together will help to advance our understanding of the human cortex as an adaptive and plastic entity that is shaped both by genetics and by its interaction with the environment.

## Methods

### Participants

A total of 3,696 subjects with available and sufficient quality MRI scans from 5 cohorts were analysed. We removed non-European descents and related individuals. The combined sample of five cohorts is made of 605 subjects from the Thematically Organized Psychosis study (mean age: 35 years, range=17–70 years)^36^, 842 Health Study of Nord-Trøndelag (HUNT) subjects (mean age: 58 years, range=50–66 years)^37^, 325 Norwegian Cognitive NeuroGenetics subjects (mean age: 52 years, range=19–79 years)^38^, 726 Alzheimer's Disease Neuroimaging Initiative subjects (mean age: 75 years, range=55–92 years) and 1,198 Pediatric Imaging Neurocognition and Genetics subjects (mean age: 12 years, range=3–21 years)^39^,^40^ (see Supplementary Methods for more details).

The samples for the twin analysis was part of the Vietnam Era Twin Study of Aging (VETSA) study^41^. There were 466 participants at age 51–60 years, of which 99 pairs were dizygotic twins and 134 pairs monozygotic twins. The sample is representative of US middle-aged men in their lifestyle and health characteristics.

Each study was approved by the local Institutional Review Board: South East Norway (Thematically Organized Psychosis and Norwegian Cognitive NeuroGenetics) and Mid Norway (HUNT) Regional Ethical Committee (HUNT), and UC San Diego (Paediatric Imaging Neurocognition and Genetics and VETSA).

### Genotype quality control and imputation

All studies were genotyped using different commercial arrays. Standard genome-wide association quality control measures were applied to each study individually using the Plink toolset^42^,^43^. Samples missing >5% of SNPs, with a minor allele frequency of <1%, or failing a test of Hardy–Weinberg equilibrium (*P*<1 × 10^−6^), were excluded. Individual samples showing an over- or underabundance of heterozygosity (>5 s.d. from the mean) were labelled as poor quality and also excluded from subsequent analyses^44^. Furthermore, to ensure that all individuals were unrelated, functions available in the software package GCTA^45^ were used to estimate kinship values from SNP genotypes for all pairs of individuals in the combined cohort. Population stratification and ancestry were assessed against a reference sample consisting of individuals from the HapMap III^46^ and 1000 Genomes^47^ via principal component analysis implemented in the software package EIGENSOFT^48^. One half of each pair of individuals with an estimated relatedness >0.025 or 0.1 was excluded^11^. Using the more stringent threshold of 0.025, 575 individuals were removed, leaving a total of 2,364 individuals for the subsequent analyses. In this combined cohort of European ancestry with minimal relatedness between subjects (GRM<0.025), 52% of the individuals were female; the subjects were aged 47±24 years (range=3–90 years); and 273, 128, 131, 147 and 66 subjects were diagnosed with mild cognitive impairment, Alzheimer's disease, schizophrenia, bipolar disorder and other psychotic, respectively. For the less stringent threshold of the estimated relatedness of 0.1, 241 individuals were removed, leaving a total of 2,698 individuals for the subsequent analyses (GRM<0.1). To maximize information present in the data and allow for comparison across multiple samples genotyped on different platforms, genotype imputation was performed using the software packages MaCH^49^ and Minimac^50^. A quality control metric (*r*^2^) was provided by Minimac and a threshold of *r*^2^>0.5 was used to declare successful imputation.

### MRI processing

MRI scans were analysed with software developed at the University of California, San Diego, Multi-Modal Imaging Laboratory based on the freely available FreeSurfer software package (http://freesurfer-software.org/). The cortical surface was reconstructed to measure surface areas at each surface location (a total of over 160,000 locations for each hemisphere) using a semi-automated approach^15^,^51^. Variation in image intensity due to magnetic field inhomogeneities was corrected, a normalized intensity image was created and the skull (non-brain) was removed from this image. The resulting surface was covered with a polygonal tessellation and smoothed to reduce metric distortions. A refinement procedure was then applied to obtain a representation of the grey/white boundary, and the resulting surface was subsequently deformed outwards to obtain an explicit representation of the pial surface. Once generated, the cortical surface model was individually reviewed, and ∼90 subjects were removed for failed surface reconstruction, often related to excessive head motion artefact. Quality control includes assessing the accuracy of surface reconstruction and subcortical segmentation, and detecting large-scale brain atrophy. In addition, manual editing was performed for technical accuracy in Alzheimer's Disease Neuroimaging Initiative and VETSA. The edits typically involve the addition or removal of voxels from white matter or brain masks to correct white matter over- or underestimation or to remove non-brain tissues labelled as grey matter. For example, white matter abnormalities commonly seen in aging populations could cause white matter incorrectly labelled as cortical surface. However, we generally find that analysis outcomes are quite similar with or without manually editing, especially in a large set of data. Maps were spatially smoothed and placed into a common coordinate system using a non-rigid high-dimensional spherical averaging method to align cortical folding patterns^51^. Due to the standardized procedure for image acquisition and analysis, the MRI data consistency was maximized for pooling the data across studies.

### Genetically based cortical phenotypes

We previously used a data-driven fuzzy clustering technique to identify parcels of the human cortex that are maximally genetically correlated (that is, under the control of similar genetic factors) based on the MRI scans of over 400 twins^6^,^16^. We used this twin-based cluster map to assign a parcellation label to each location on a cortical surface based on partial membership information estimated from the clustering analysis, and calculated the weighted average surface area within each region for each subject. We used these regions, defined *a priori* on the basis of genetic information, to increase power for detecting effects and minimize multiple comparisons after reducing image dimensionality to these parcels.

To account for global effects, we divided the area measure of each location by the total surface area, so that the observed effects were specific to region of interest rather than having global associations with total surface area^6^,^16^. In the present study, in all analyses we further adjusted each phenotype for the covariates of age, gender, age × gender, scanner, diagnosis and study cohort. The sample age ranges from 3 to 90 years. We used polynomial basis functions and a generalized additive modelling framework to model age effects as nonparametric smooth functions to control for considerable age disparities^52^, so the effects that we characterized can be generalized across the lifespan.

### SNP heritability

We used a mixed linear model to fit a covariance structure of GRM to a vector of measured phenotypes via the GCTA tool^9^,^11^ to estimate the proportion of phenotypic variance captured by all autosomal SNPs. The analyses included common SNPs (for example, minor allele frequency >0.01). We included the top 10 eigenvectors of the principal component analysis of an allele-sharing distance matrix or GRM as covariates to capture any remaining population structure in our European data set.

As described further in the Supplementary Methods, the mixed linear model analysis in quantitative genetics partitions the phenotypic variance–covariance matrix between two (or more) specified matrices. One typical form is:


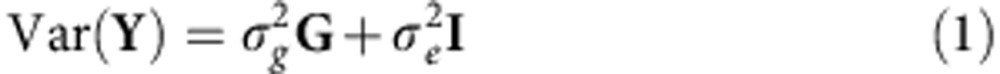


where G is a matrix of kinship or genetic correlation coefficients and I is the *n* × *n* identity matrix, which assumes independence of environmental effects (that is, no shared environment) and measurement error across individuals. Estimates 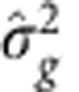 and 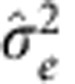 are typically obtained via restricted maximum likelihood. Narrow-sense heritability, 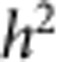, the proportion of phenotypic variance explained by additive genetic effects, is estimated by


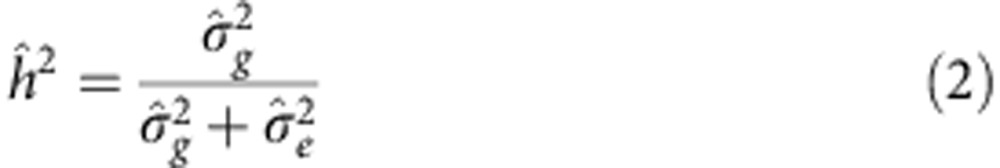


The resulting estimate is referred to as ‘SNP' or ‘chip heritability' (*h*^2^), since it is based on the SNPs used to construct the GRM^12^,^13^.

### Partitioning of genomic variation by genic annotation

We partitioned the variance explained by all of the SNPs into genic and intergenic regions of the whole genome. We obtained 24,526 gene boundaries from the UCSC Genome Browser hg19 assembly. We defined genic boundaries as ±20 kb upstream and downstream from the 3′ and 5′ UTRs of each gene, where genic and intergenic coverages are roughly equal (∼50%). This definition was used previously^17^. We estimated the proportion of variance explained by genic and intergenic regions. The results are shown in Fig. 2a and Supplementary Table 4 for the GRM<0.025-sample and Supplementary Fig. 2a and Supplementary Table 7 for the GRM<0.1-sample. We further used an LD-weighted genic annotation scheme that takes into account the LD structure to select SNPs that are related to exon, intron, 3′ UTR, 5′ UTR and 1 kb upstream and downstream of genes (six genic categories) (see Supplementary Methods).

### Partitioning of genomic variation by conservation annotation

We partitioned the variance explained by all of the SNPs into low- and high-conserved regions of the whole genome based on conservation annotation. We obtained a conservation annotation database from the UCSC Genome Browser hg19 assembly. The conservation scores were derived from alignments of placental mammals to human genome. PhastCons is a hidden Markov model-based method that estimates the probability that each nucleotide belongs to a conserved element, based on the multiple alignments^19^.

We assigned weights to conservation scores based on the LD information. We applied the pairwise LD matrix to the vector of phastCons scores. We expect that SNPs with the LD-weighted conservation annotation show more consistent and less noisy association signals. After the LD weighting, 48,523 of the ∼2.4 million SNPs had no scores and were eliminated from the subsequent analysis. We selected the median as a threshold to partition the genome evenly into low- and high-conserved SNPs (∼50%). We estimated the proportion of variance explained by low- and high-conserved genomic regions. The results are shown in Fig. 3a and Supplementary Table 6 for the GRM<0.025-sample, and Supplementary Fig. 3 and Supplementary Table 9 for the GRM<0.1-sample.

## Additional information

**How to cite this article:** Chen, C.-H. *et al*. Large-scale genomics unveil polygenic architecture of human cortical surface area. *Nat. Commun.* 6:7549 doi: 10.1038/ncomms8549 (2015).

## Supplementary Material

Supplementary Information Supplementary Figures 1-3, Supplementary Tables 1-9, Supplementary Methods and Supplementary References
