# Supplementary material for: Large-scale genomics unveil polygenic architecture of human cortical surface area
Source: Nat Commun. 2015 Jul 20;6:7549. doi: 10.1038/ncomms8549 (PMC4518289; doi:10.1038/ncomms8549)
Supplement: Supplementary Information — Supplementary Figures 1-3, Supplementary Tables 1-9, Supplementary Methods and Supplementary References [file ncomms8549-s1.pdf]

Supplementary Fig. 1: A comparison of estimates of variance explained by SNPs with two relatedness thresholds and with the LD adjusted genetic relationships

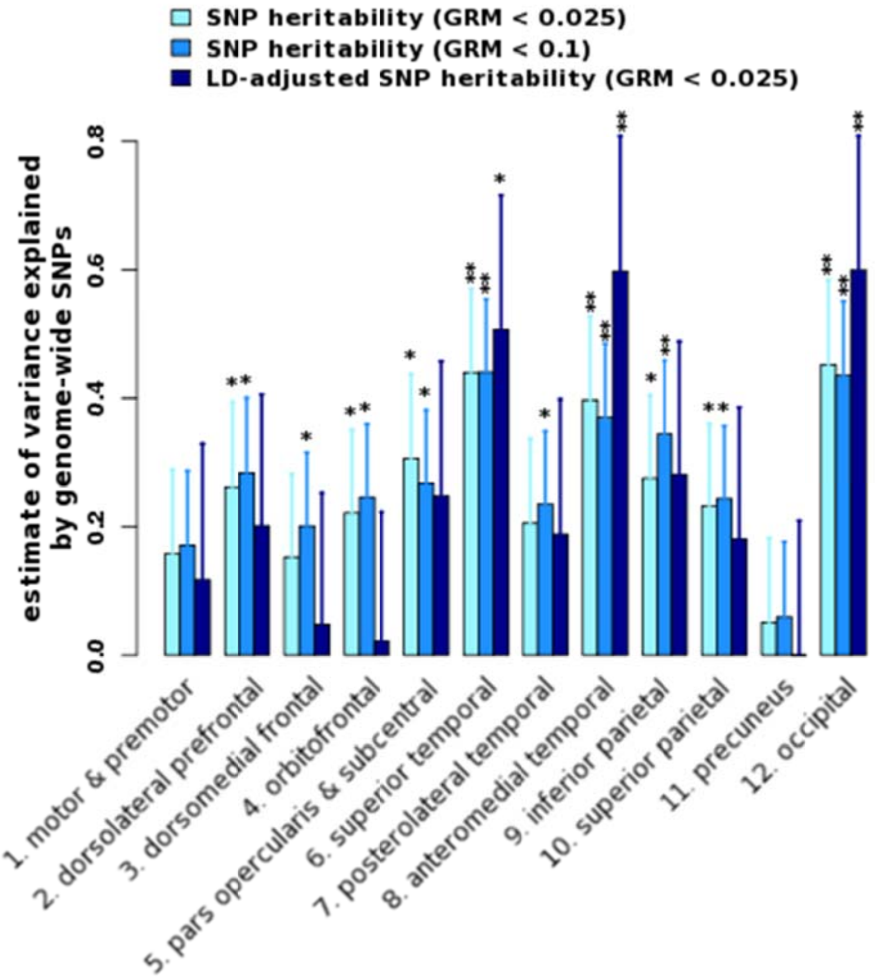

Estimates of variance explained by all autosomal SNPs from genetic relationships less than 0.025 or 0.1 (GRM<0.025) or (GRM<0.1), respectively. Estimates of variance explained by all autosomal SNPs from GRM < 0.025 with adjusting the genetic relationships by the linkage disequilibrium structure. Error bars represent the standard errors of the estimates.

Supplementary Fig. 2: Partitioning of genomic variation by genic annotation (GRM < 0.1) (Fig. 2 in the text was based on GRM < 0.025)

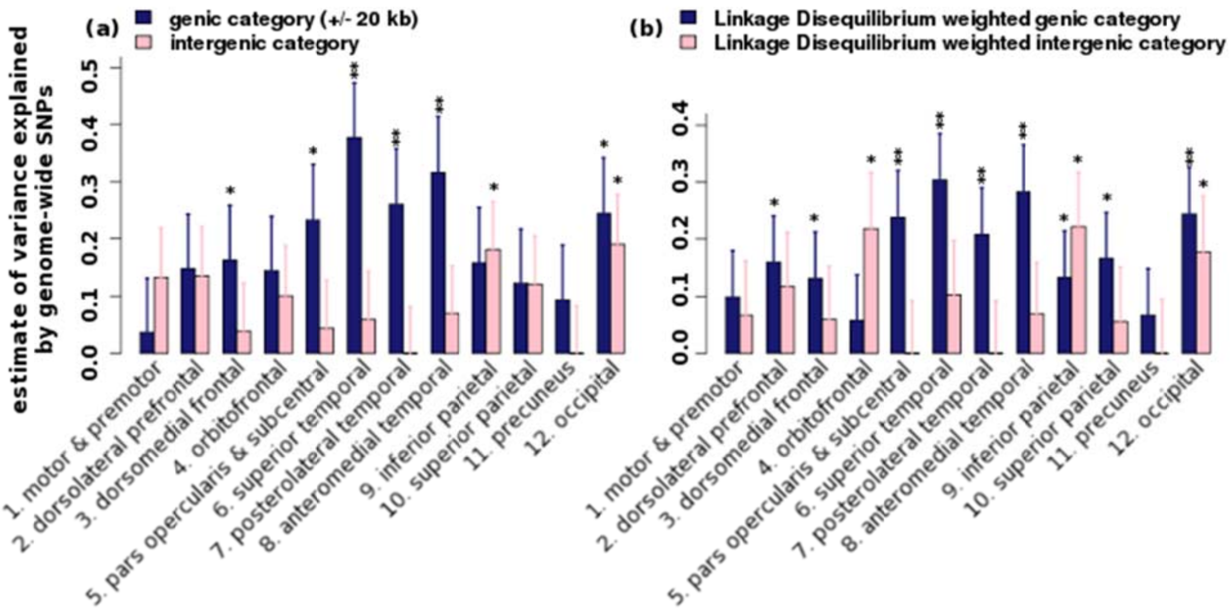

(a) Estimates of variance explained by genic and intergenic regions (GRM <0.1). The genic region is defined as ±20kb from the 3' and 5' UTRs. (b) Estimates of variance explained by genic and intergenic regions. The genic region is defined by the LD-weighted genic annotation scheme. * $p < 0.05$, ** $p < 0.004$. Error bars represent the standard errors of the estimates.

Supplementary Fig. 3: Partitioning of genomic variation by conservation annotation (GRM < 0.1) (Fig. 3 in the text was based on GRM < 0.025)

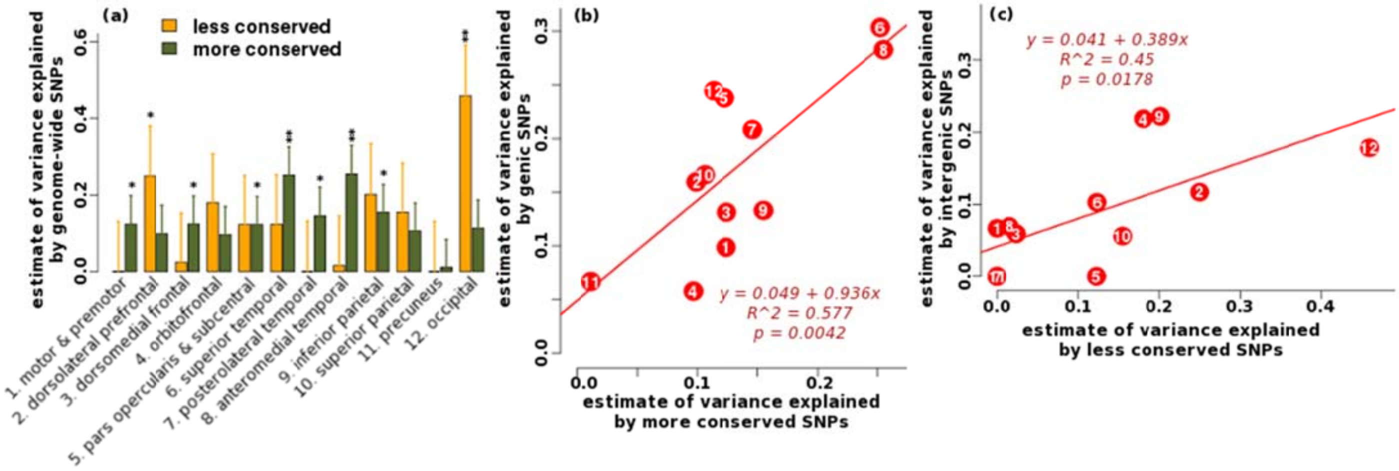

(a) Estimates of variance explained by conserved and nonconserved regions (GRM <0.1). * $p < 0.05$, ** $p < 0.004$. Error bars represent the standard errors of the estimates.

(b) A significant correlation between estimates of variance explained by genic and more conserved SNPs across phenotypes. (c) A significant correlation between estimates of variance explained by intergenic and less conserved SNPs. This relation is not tested in the GRM < 0.025 sample due to many zero values for the variance explained by less conserved SNPs (see Fig. 3).

Supplementary Table 1: A comparison of estimates of variance explained by all autosomal SNPs with two relatedness thresholds

	GRM < 0.025			GRM < 0.1		
**cortical phenotype**	**h^2**	**s.e.**	**p**	**h^2**	**s.e.**	**p**
motor_premotor	0.16	0.132	0.117	0.17	0.116	0.072
dorsolateral prefrontal	0.26	0.133	0.025*	0.28	0.116	7.6e-03*
dorsomedial frontal	0.15	0.130	0.119	0.20	0.115	0.039*
orbitofrontal	0.22	0.130	0.042	0.25	0.114	0.015*
pars opercularis & subcentral	0.31	0.131	9.2e-03*	0.27	0.114	8.5e-03*
superior temporal	0.42	0.131	3.4e-04**	0.44	0.114	3.8e-05**
posterolateral temporal	0.21	0.132	0.060	0.23	0.114	0.018*
anteromedial temporal	0.40	0.130	8.9e-04**	0.37	0.114	4.5e-04**
inferior parietal	0.28	0.129	0.014*	0.34	0.114	9.9e-04**
superior parietal	0.23	0.129	0.033*	0.24	0.114	0.014*
precuneus	0.05	0.131	0.348	0.06	0.117	0.309
occipital	0.45	0.132	2.9e-04**	0.44	0.116	8.3e-05**

Estimates of variance explained by all autosomal SNPs from genetic relationships less than 0.025 or 0.1 (GRM<0.025) or (GRM<0.1), respectively. s.e.: standard error. Estimates were tested for significantly different from zero by likelihood ratio test.

Supplementary Table 2: A comparison of estimates of variance explained by all autosomal SNPs with and without LD adjusted genetic relationships

	GRM < 0.025			LD-Adjusted GRM < 0.025		
**cortical phenotype**	**h^2**	**s.e.**	**p**	**h^2**	**s.e.**	**p**
motor_premotor	0.16	0.132	0.117	0.12	0.212	0.293
dorsolateral prefrontal	0.26	0.133	0.025*	0.20	0.204	0.158
dorsomedial frontal	0.15	0.130	0.119	0.05	0.205	0.406
orbitofrontal	0.22	0.130	0.042*	0.02	0.201	0.456
pars opercularis & subcentral	0.31	0.131	9.2e-03*	0.25	0.210	0.121
superior temporal	0.42	0.131	3.4e-04**	0.51	0.209	8.1e-03*
posterolateral temporal	0.21	0.132	0.060	0.19	0.210	0.187
anteromedial temporal	0.40	0.130	8.9e-04**	0.60	0.210	2.6e-03**
inferior parietal	0.28	0.129	0.014*	0.28	0.207	0.086
superior parietal	0.23	0.129	0.033*	0.18	0.205	0.187
precuneus	0.05	0.131	0.348	0.00	0.209	0.5
occipital	0.45	0.132	2.9e-04**	0.60	0.209	2.3e-03**

Estimates of variance explained by all autosomal SNPs from GRM < 0.025 with and without adjusting the genetic relationships by the linkage disequilibrium structure. The adjustment was implemented in LDAK (Linkage-Disequilibrium Adjusted Kinships). Estimates were tested for significantly different from zero by likelihood ratio test.

Supplementary Table 3: Twin heritability estimates

cortical phenotype	rMZ	rDZ	Variance components							p-values		
			a^2	95% CI	c^2	95% CI	e^2	95% CI	-2LL	no A	no C	no AC
motor premotor	0.74	0.36	0.74	(0.45, 0.80)	0.00	(0, 0.27)	0.26	(0.20, 0.34)	1203.81	< 0.0001	1.00	< 0.0001
dorsolateral PFC	0.57	0.36	0.45	(0.08, 0.66)	0.12	(0, 0.44)	0.43	(0.34, 0.55)	1255.81	0.02	0.51	< 0.0001
dorsomedial frontal	0.63	0.39	0.58	(0.25, 0.73)	0.07	(0, 0.36)	0.35	(0.27, 0.46)	1238.54	0.0006	0.68	< 0.0001
orbitofrontal	0.59	0.36	0.43	(0.07, 0.67)	0.15	(0, 0.46)	0.42	(0.33, 0.54)	1250.33	0.02	0.43	< 0.0001
pars oper. & subcentral	0.61	0.33	0.57	(0.20, 0.70)	0.04	(0, 0.36)	0.39	(0.30, 0.51)	1249.67	0.02	0.81	< 0.0001
superior temporal	0.57	0.21	0.58	(0.36, 0.68)	0.00	(0, 0.18)	0.42	(0.32, 0.54)	1265.48	< 0.0001	1.00	< 0.0001
posterolateral temporal	0.51	0.38	0.21	(0, 0.58)	0.29	(0, 0.54)	0.51	(0.40, 0.63)	1266.15	0.30	0.14	< 0.0001
anteromedial temporal	0.61	0.28	0.58	(0.20, 0.67)	0.00	(0, 0.34)	0.42	(0.33, 0.53)	1253.37	0.003	1.00	< 0.0001
inferior parietal	0.67	0.29	0.67	(0.44, 0.75)	0.00	(0, 0.20)	0.33	(0.25, 0.43)	1235.95	< 0.0001	1.00	< 0.0001
superior parietal	0.66	0.17	0.63	(0.45, 0.72)	0.00	(0, 0.15)	0.37	(0.28, 0.48)	1246.76	< 0.0001	1.00	< 0.0001
precunues	0.55	0.29	0.51	(0.12, 0.65)	0.04	(0, 0.37)	0.45	(0.35, 0.58)	1264.47	0.01	0.85	< 0.0001
occipital	0.70	0.43	0.59	(0.29, 0.78)	0.12	(0, 0.39)	0.29	(0.22, 0.38)	1209.62	< 0.0001	0.46	< 0.0001

rMZ: monozyotic correlation, rDZ: dizygotic correlation, a^2 = additive genetic variance (heritability estimates), c^2 = shared environmental variance, e^2 = unique, unshared environmental variance, CI: confidence interval, −2LL = Negative 2 log-likelihood for the ACE model, no A: test of CE model, i.e., hypothesis of no additive genetic effect; no C: test of AE model, i.e., hypothesis of no shared environmental effects; no AC: test of E-only model, i.e., hypothesis of no additive genetic and common environmental effects. The analyses were adjusted for age, MRI scanners, and total surface area.

Supplementary Table 4: Partitioning of genomic variation by genic annotation (±20kb)(GRM < 0.025)

	genic (±20kb)			intergenic		
**cortical phenotype**	h_g^2	**s.e.**	**p**	h_i^2	**s.e.**	**p**
motor_premotor	0.07	0.107	0.225	0.08	0.097	0.212
dorsolateral prefrontal	0.12	0.108	0.125	0.14	0.097	0.074
dorsomedial frontal	0.11	0.108	0.134	0.06	0.094	0.341
orbitofrontal	0.00	0.106	0.339	0.17	0.099	0.035*
pars opercularis & subcentral	0.26	0.112	0.012*	0.06	0.096	0.261
superior temporal	0.32	0.108	4.8e-04**	0.10	0.096	0.161
posterolateral temporal	0.20	0.113	7.7e-03*	0.00	0.093	0.5
anteromedial temporal	0.20	0.110	1.5e-03**	0.08	0.094	0.172
inferior parietal	0.06	0.109	0.239	0.19	0.096	0.021*
superior parietal	0.14	0.109	0.066	0.10	0.095	0.212
precuneus	0.08	0.107	0.204	0.00	0.094	0.5
occipital	0.19	0.111	0.022*	0.27	0.097	9.7e-03*

Estimates of variance explained by genic and intergenic regions (GRM <0.025). The genic region is defined as ±20kb from the 3' and 5' UTRs. s.e.: standard error. Estimates were tested for significantly different from zero by likelihood ratio test.

Supplementary Table 5: Partitioning of genomic variation by LD-weighted genic annotation (GRM < 0.025)

	LD-weighted genic			LD-weighted intergenic		
**cortical phenotype**	h_g^2	**s.e.**	**p**	h_i^2	**s.e.**	**p**
motor_premotor	0.11	0.091	0.100	0.03	0.107	0.403
dorsolateral prefrontal	0.14	0.092	0.061	0.12	0.107	0.131
dorsomedial frontal	0.09	0.092	0.151	0.05	0.105	0.303
orbitofrontal	0.00	0.088	0.5	0.28	0.110	6.1-e03*
pars opercularis & subcentral	0.25	0.094	3.2-e03**	0.03	0.105	0.399
superior temporal	0.29	0.093	8.6e-04**	0.13	0.107	0.109
posterolateral temporal	0.19	0.093	0.014*	0.00	0.105	0.5
anteromedial temporal	0.27	0.093	1.3-e03**	0.11	0.103	0.139
inferior parietal	0.07	0.090	0.230	0.23	0.107	0.013*
superior parietal	0.14	0.092	0.057	0.08	0.107	0.229
precuneus	0.05	0.091	0.241	0.00	0.106	0.5
occipital	0.21	0.094	0.012*	0.25	0.111	0.015*

Estimates of variance explained by genic and intergenic regions. The genic region is defined by the LD-weighted genic annotation scheme. s.e.: standard error. Estimates were tested for significantly different from zero by likelihood ratio test.

Supplementary Table 6: Partitioning of genomic variation by conservation annotation (GRM < 0.025)

	less conserved			more conserved		
**cortical phenotype**	h_1^2	**s.e.**	**p**	h_2^2	**s.e.**	**p**
motor_premotor	0.00	0.152	0.5	0.09	0.083	0.048*
dorsolateral prefrontal	0.24	0.149	0.050*	0.09	0.084	0.141
dorsomedial frontal	0.00	0.149	0.5	0.09	0.084	0.097
orbitofrontal	0.12	0.143	0.198	0.10	0.083	0.105
pars opercularis & subcentral	0.00	0.149	0.489	0.20	0.085	8.9e-03*
superior temporal	0.00	0.148	0.5	0.30	0.083	8.7e-05**
posterolateral temporal	0.00	0.152	0.5	0.12	0.085	0.064
anteromedial temporal	0.00	0.149	0.5	0.27	0.084	3.0e-04**
inferior parietal	0.17	0.149	0.128	0.12	0.081	0.065
superior parietal	0.18	0.145	0.097	0.08	0.083	0.160
precuneus	0.00	0.154	0.5	0.08	0.087	0.5
occipital	0.46	0.150	1.1e-03**	0.13	0.083	0.060

Estimates of variance explained by less conserved and more conserved regions. s.e.: standard error. Estimates were tested for significantly different from zero by likelihood ratio test.

Supplementary Table 7: Partitioning of genomic variation by genic annotation (±20kb)(GRM < 0.1)

	genic (±20kb)			intergenic		
**cortical phenotype**	h_g^2	**s.e.**	**p**	h_i^2	**s.e.**	**p**
motor_premotor	0.04	0.094	0.349	0.13	0.086	0.062
dorsolateral prefrontal	0.15	0.095	0.054	0.14	0.086	0.055
dorsomedial frontal	0.16	0.095	0.039*	0.04	0.084	0.322
orbitofrontal	0.14	0.095	0.060	0.10	0.087	0.129
pars opercularis & subcentral	0.23	0.097	8.6e-03*	0.04	0.084	0.302
superior temporal	0.38	0.095	2.0e-05**	0.06	0.084	0.244
posterolateral temporal	0.26	0.097	2.8e-03**	0.00	0.082	0.5
anteromedial temporal	0.32	0.097	5.5e-04**	0.07	0.082	0.191
inferior parietal	0.16	0.097	0.051	0.18	0.084	0.013*
superior parietal	0.12	0.094	0.092	0.12	0.085	0.074
precuneus	0.09	0.096	0.132	0.00	0.083	0.5
occipital	0.24	0.097	5.8e-03*	0.19	0.086	0.012*

Estimates of variance explained by genic and intergenic regions (GRM <0.1). The genic region is defined as ±20kb from the 3' and 5' UTRs. s.e.: standard error. Estimates were tested for significantly different from zero by likelihood ratio test.

Supplementary Table 8: Partitioning of genomic variation by LD-weighted genic annotation (GRM < 0.1)

	LD-weighted genic			LD-weighted intergenic		
**cortical phenotype**	h_g^2	**s.e.**	**p**	h_i^2	**s.e.**	**p**
motor_premotor	0.10	0.081	0.109	0.07	0.095	0.245
dorsolateral prefrontal	0.16	0.081	0.021*	0.12	0.095	0.107
dorsomedial frontal	0.13	0.081	0.049*	0.06	0.093	0.262
orbitofrontal	0.06	0.079	0.230	0.22	0.098	0.014*
pars opercularis & subcentral	0.24	0.082	1.5e-0.3**	0.00	0.092	0.5
superior temporal	0.30	0.081	5.7e-05**	0.10	0.094	0.138
posterolateral temporal	0.21	0.081	3.4e-03**	0.00	0.091	0.5
anteromedial temporal	0.28	0.082	2.1-e0.4**	0.07	0.090	0.212
inferior parietal	0.13	0.081	0.048*	0.22	0.094	0.008*
superior parietal	0.17	0.080	0.016*	0.06	0.095	0.282
precuneus	0.07	0.081	0.143	0.00	0.094	0.5
occipital	0.24	0.082	1.3e-03**	0.18	0.098	0.037*

Estimates of variance explained by genic and intergenic regions. The genic region is defined by the LD-weighted genic annotation scheme. s.e.: standard error. Estimates were tested for significantly different from zero by likelihood ratio test.

Supplementary Table 9: Partitioning of genomic variation by conservation annotation (GRM < 0.1)

	less conserved			more conserved		
**cortical phenotype**	h_1^2	**s.e.**	**p**	h_2^2	**s.e.**	**p**
motor_premotor	0.00	0.131	0.5	0.12	0.075	0.014*
dorsolateral prefrontal	0.25	0.130	0.025*	0.10	0.074	0.090
dorsomedial frontal	0.02	0.128	0.424	0.12	0.073	0.042*
orbitofrontal	0.18	0.127	0.070	0.10	0.073	0.089
pars opercularis & subcentral	0.12	0.129	0.167	0.12	0.073	0.044*
superior temporal	0.12	0.129	0.170	0.25	0.073	1.5e-0.4**
posterolateral temporal	0.00	0.132	0.5	0.15	0.075	0.021*
anteromedial temporal	0.02	0.130	0.453	0.25	0.074	2.1e-0.4**
inferior parietal	0.20	0.132	0.065	0.16	0.072	0.012*
superior parietal	0.15	0.129	0.111	0.11	0.073	0.070
precuneus	0.00	0.131	0.5	0.01	0.072	0.276
occipital	0.46	0.131	2.5e-0.4**	0.11	0.073	0.057

Estimates of variance explained by less conserved and more conserved regions. s.e.: standard error. Estimates were tested for significantly different from zero by likelihood ratio test.

Supplemental Methods

Participants

Thematically Organized Psychosis (TOP) Subjects. Data from 1579 subjects from the TOP study were analyzed. MRI data was available for 605 of the subjects, including 236 controls, 144 subjects with schizophrenia (SCZ), 157 subjects with bipolar disorder (BIP), and 68 subjects diagnosed with other psychotic (OP) disorders not otherwise specified. Fifty percent of the subjects were women; the subjects were aged 35 ± 11 y (range = 17–70 y). Genotyping: DNA was genotyped on the Affymetrix 6.0 array. All subjects self-reported Norwegian ancestry. PCA of an allele-sharing distance matrix across all subjects suggested one non-European ancestry genetic outlier. Brain imaging: MRI scans were performed with a 1.5 T Siemens Magnetom Sonata scanner equipped with a standard head coil.

Health Study of Nord-Trøndelag (HUNT) Subjects. Data from 905 subjects from the HUNT study were analyzed. Cortical MRI data was available for 842 of the subjects. Fifty-three percent of the subjects were women; the subjects were aged 58 ± 4 y (range = 50–66 y). Genotyping: DNA was genotyped on the Illumina Omni 2.5M BeanChip array. All subjects were part of a general population based survey, the HUNT study and recruited from one country in Norway. PCA of an allele-sharing distance matrix across all subjects suggested 2 non-European ancestry genetic outliers. Brain imaging: MRI scans were performed with a General Electric Signa HDx 1.5 T scanner.

Norwegian Cognitive NeuroGenetics (NCNG) Subjects. Data from 670 subjects from the NCNG study were analyzed. MRI and genetic data were available for 325 of the subjects. Sixty-nine percent of the subjects were women; the subjects were aged 52 ± 17 y (range = 19–79 y). Genotyping: DNA was genotyped with the Illumina Human610-Quad BeadChip. All subjects self-reported Norwegian ancestry. PCA of an allele-sharing distance matrix across all subjects did not suggest any non-European ancestry genetic outliers. Brain imaging: Participants went through a standard structural MRI protocol optimized for morphometric analysis. Further details about MRI acquisition and MRI protocols are available in Espeseth et al., 2012.[1]

Pediatric, Imaging, Neurocognition, and Genetics (PING) Subjects. Data from 1406 subjects were obtained from the PING database (http://ping.chd.ucsd.edu/). MRI data from 1198 subjects were included. The subjects were aged 12 ± 5 y (range = 3-21 y) and 48% of the subjects were female. Genotyping: DNA was genotyped with the Illumina Human660W-Quad BeadChip; Based on principal component analysis of an allele-sharing distance matrix, 668 individuals were removed as non-European ancestry genetic outliers. Brain Imaging: T1-weighted MRI data were collected on 3-T scanners at nine study centers across the United States. Specific MRI scanner protocols are available at the PING study website (http://ping.chd.ucsd.edu/).

Alzheimer's Disease Neuroimaging Initiative (ADNI) Subjects. Data from 793 subjects from the ADNI database were analyzed (http://adni.loni.usc.edu); Usable MRI data was available for 726 of the subjects. Subjects who self-reported as white and non-Hispanic

included 161 individuals with Alzheimer's Disease (AD), 216 individuals with Mild Cognitive Impairment (MCI), and 216 subjects as controls. Forty-three percent of the subjects were women; the subjects were aged 75 ± 7 y (range = 55-92 y). Genotyping: DNA was genotyped with the Illumina Human610-Quad BeadChip. PCA of an allele-sharing distance matrix was used to remove 85 individuals as non-European ancestry genetic outliers. Brain Imaging: MRI data were collected on 1.5-T scanners at many study centers across the United States. Raw MR images were downloaded from the ADNI data page of the public ADNI site at the LONI website published in 2007.

We estimated the effects of scanners and study sites on our phenotypes. To estimate the percentage of phenotypic variation uniquely explained by scanner types and study sites, phenotype data were first residualized for other covariates (e.g., age and gender). The estimated adjusted R square for scanners and study sites are small (0.014 and 0 respectively, averaged across all 12 cortical regions).

Mixed linear model to estimate the variance explained by all autosomal SNPs

The mixed linear model analysis in quantitative genetics partitions the phenotypic variance-covariance matrix between two (or more) specified matrices. One typical form is:

$$\mathrm{Var}(\mathbf{Y}) = \sigma_g^2\mathbf{G} + \sigma_e^2\mathbf{I} \qquad (1)$$

where G is a matrix of kinship or genetic correlation coefficients and I is the n x n identity matrix, which assumes independence of environmental effects (i.e., no shared environment) and measurement error across individuals. Estimates $\hat{\sigma}_g^2$ and $\hat{\sigma}_e^2$ are typically obtained via restricted maximum likelihood (REML). Narrow-sense heritability, h^2, the proportion of phenotypic variance explained by additive genetic effects, is estimated by

$$\hat{h}^2 = \frac{\hat{\sigma}_g^2}{\hat{\sigma}_g^2 + \hat{\sigma}_e^2} \qquad (2)$$

If we can genotype subjects at causal variants, we include all causal variants in the model[2]

$$\mathbf{y}_j = \boldsymbol{\mu} + \mathbf{g}_j + \mathbf{e}_j \text{ and } \mathbf{g} = \textstyle\sum_{i=1}^{m} \mathbf{Z}_{ji}\mathbf{u}_i \qquad (3)$$

where g_j is the total genetic effect of an individual j, m is the number of causal loci, and u_i is the scaled additive genetic effect of the ith causal variant; Z_{ji} is the genotypic values standardized across all subjects. Genotypic values are 0,1, or 2 if the genotype of the jth individual at SNP i is bb, Bb, or BB. If Z were not column standardized, Equation 2 might not hold.[3] u_i and e_j are independently Gaussian with zero mean and variance σ_g^2/m and σ_e^2, respectively. E(Y) = μ, and $\mathrm{Var(Y)} = \frac{\sigma_g^2}{\mathrm{m}}\mathrm{ZZ^T} + \sigma_e^2\mathrm{I}$. Thus, we have Equation 1 with $\frac{\mathrm{ZZ^T}}{\mathrm{m}}$ in place of G. However, we know little about what the causal variants are, so $\frac{\mathrm{ZZ^T}}{\mathrm{m}}$ is unknown. Yang et al. developed a method with which to calculate the approximation of G from genome-wide SNP genotypes.[2] Thus, we have Equation 1 with $\mathrm{A} = \frac{\mathrm{XX^T}}{\mathrm{m'}}$ in place of G, where X is defined in the same way as Z with the exception that SNP genotypes replace causal variant genotypes and m′ is the number of SNPs.[2,3]

The general mixed model has long been a foundation of both theory and application in the study of quantitative genetics.[4,5] The term 'mixed' refers to the presence of both a random effect of total genetic variance (g), usually interpreted in terms of a polygenic contribution to the given trait, and fixed effects corresponding to covariates or individual SNPs.[3,6,7]

Before dense genome-wide genetic markers became widely available in recent years, the elements of the matrix G were coefficients derived from the probabilities of identity-by-descent on the basis of the recorded pedigree, representing expected genetic relatedness between relatives. For example, in the classical twin modeling, 1 represented monozygotic twin pairs and 0.5 dizygotic twin pairs. Therefore, both the SNP and twin heritability are rooted in variance component analysis and the mixed model to estimate genetic effects in the aggregate, providing the rationale for comparing them in this paper.

Simulation

We performed simulation studies based on the observed genotype data of our five sub-study combined sample (~2.4 million imputed SNPs). We randomly sampled m SNPs as causal variants and generated the effect of each causal variant (b) from a standard normal distribution. We calculated the genetic value of each individual by $g = \sum x_i b_i$, where x is coded as 0, 1, or 2 for genotype bb, Bb, or BB, respectively, at the locus i. We generated residual effects (e) from N (0, var(g) $(1-h^2)/h^2$) and produced the simulated phenotype by y = g + e. We used nine predefined heritability values (h^2= 0.1 - 0.9, with 0.1 interval) and different parameter settings including: (1) different proportions of causal variants (e.g., 0.04%, 1%, and 3%); (2) allowing mixture distribution for causal variants with large and small effects (e.g., proportion of large effects = 1, 0.8, or 0.2); (3) randomly pruning SNPs at the threshold of r^2 = 0.9 (i.e., randomly excluding one of each pair of SNPs with high linkage disequilibrium (LD)), and retaining 795,706 imputed genome-wide SNPs. The genome-wide LD values were calculated based on the 1000 Genomes Project data.[8] For each setting, we repeated the simulation 100 times, randomizing the positions of causal variants in each simulation replicate.

We performed heritability estimation of the simulated phenotypic and observed genotype data in GCTA. In all scenarios, the simulation showed small differences between estimated and true/predefined h^2. The average differences (across 100 simulations and across 9 heritability values) ranged from 0.037 to 0.046 for different parameter settings, suggesting that the method is reasonably robust to apply to our sample in various scenarios of genetic architecture. Our findings from the simulation are consistent with other studies confirming the reliability of the method.[3,9]

Power Calculation

Given sample size and heritability of cortical regions, power calculations were performed using the power calculator implemented in GCTA.[10] We calculated the probability of detecting $h^2 > 0$ for the Type 1 error at a threshold of 0.05. Because the true SNP heritability in the population was unknown, we used the heritability estimated from the twin sample (Supplementary Table 3). Note that twin heritability estimates are considered

as the upper bound of the SNP heritability, so the estimated power here is regarded as maximum. The variance of the SNP-derived genetic relationship was 2.35e-05 in our sample. We calculated the power to detect heritability for each cortical phenotype: Motor-premotor cortex (0.9928), occipital cortex (0.9979), posterolateral temporal cortex (0.2318), superior parietal cortex (0.9749), orbitofrontal cortex (0.9862), superior temporal cortex (0.9173), inferior parietal cortex (0.9487), dorsomedial frontal cortex (0.3122), anteromedial temporal cortex (0.7927), precuneus (0.0508), dorsolateral prefrontal cortex (0.7927), and subcentral region (0.8734).

The effect of patient samples

Although all of the phenotypes were adjusted for patient diagnosis prior to model fitting in all analyses, we examined any effect of patient samples on our results. Including individuals with diseases (all subjects) offered the advantage of a larger sample size and wider phenotype distribution, which may provide greater power for reliable estimation.[11] To examine the effects of patient samples on our results, we reanalyzed heritability estimation after we eliminated one disorder at a time (MCI, AD, SCZ, BIP, and OP). Each of these five secondary analyses gave us very similar results. The SNP heritability estimates were highly correlated between the full combined sample and the combined sample without one patient group. Here we listed the correlation coefficients for each comparison: without MCI: $r = 0.92$, $p = 2.28\text{e-}05$; without AD: $r = 0.94$, $p = 5.77\text{e-}06$; without SCZ: $r = 0.95$, $p = 2.50\text{e-}06$; without BIP: $r = 0.93$, $p = 9.08\text{e-}06$; without OP: $r = 0.97$, $p = 2.30\text{e-}07$. The analyses confirmed that the observed associations were not due to confounds related to any of the disorders.

The effect of linkage disequilibrium (LD) structure

While the method developed by Yang et al. is robust even when some assumptions are violated, the LD structure could generate biases in the estimation of SNP heritability.[3] Contributions to heritability from causal variants tend to be overestimated in regions of strong LD and underestimated in regions of low LD. Additionally, patterns of LD are strongly linked to minor allele frequency. Low-frequency variants are likely to have lower LD with common SNPs than high-frequency variants. Therefore, heritability can be underestimated for traits with predominantly low-frequency causal variants and can be overestimated for those with predominantly high-frequency causal variants.[3] However, the standardization of genotypic values in the method (see the mixed linear model section above) implies that low-frequency variants have larger effects.[3] Thus, depending on its genetic architecture, a trait's heritability estimate could be unaffected or biased, or the biases could counteract each other. For example, underestimation of genetic effects in low-LD regions could be balanced by overestimation in high-LD regions.[3]

To examine the effect of LD on our data, we calculated the LD-adjusted kinship or genetic relationship matrix (A^*) implemented in LDAK (Linkage-Disequilibrium Adjusted Kinships).[3] We found that heritability estimates for the majority of cortical regions were not evidently biased by uneven LD structure, because the estimates remained similar (within ± 0.1). Possible exceptions were the occipital, orbitofrontal, and anteromedial temporal cortices. Additionally, the sampling variance of the SNP heritability estimates from the LD-adjusted kinship matrix was large (s.e. = ~21%). For these two reasons, we used the unadjusted genetic relationship matrix estimated from

GCTA in our main analysis. Nevertheless, the effects of LD structure and allele frequencies on precise heritability estimates in these cortical regions warrant future investigation with a larger sample size. The results from the LD-adjusted genetic relationships are shown in Supplementary Fig. 1 and Supplementary Table 2.

Twin based heritability estimation

Heritability estimates for brain imaging phenotypes from a twin sample were calculated using an ACE model implemented in OpenMx software. The twins were obtained for men aged 51-60 years enrolled in the Vietnam Era Twin Study of Aging.[12,13] In the classical twin design, the variance of each phenotype is decomposed into the proportion of total variance attributed to additive genetic (A) influences, common or shared environmental (C) influences, and unique environmental (E) influences.[14,15] The name 'ACE model' is derived from these three components of variance. Additive genetic influences are assumed to correlate 1.0 between MZ twins who generally share 100% of their genes and .5 between DZ twins who share an average of 50% of their segregating genes. Shared environment (environmental influences that make twins similar) is assumed to correlate 1.0 between both members of a twin pair, regardless of twin zygosity. Unique environmental influences (environmental influences that make twins different) are assumed to be uncorrelated between the members of a twin pair. Measurement error is also included in the E term because it is also assumed to be uncorrelated between twins. The proportion of a phenotype's total variance attributable to additive genetic influences is considered the heritability of the phenotype. The results are shown in Fig. 1(c) and Supplementary Table 3.

Partitioning of genomic variation by LD-weighted genic annotation

We used an LD-weighted genic annotation scheme that takes into account the LD structure to select SNPs that are related to exon, intron, 3'UTR, 5'UTR, and 1kb upstream and downstream of genes (six genic categories). Because the position of causal variants in the genome is unknown, a genotyped SNP can be seen as a surrogate to capture the genetic effects of causal variants in LD with the given SNP. Thus, by incorporating LD information, the annotation of individual SNPs reflects the weighted annotation in the context of underlying linkage blocks. With the LD-weighted annotation, we have shown stronger and more consistent association signals.[8] As previously described, we consistently found that SNPs capturing variants in coding and regulatory elements of genes are more enriched for association than other annotation categories across diverse phenotypes. It is worth noting that this pattern is not confounded by total LD.[8] Thus, it is not the LD structure per se that contributes to enrichment association but the annotation incorporated with LD information.

We used GWAS summary statistics for height to combine the LD-weighted annotation scores of the six genic categories into one score for each SNP. Height, considered as a generic trait of body size, is highly polygenic and heritable. SNP associations with height have been shown to have implications for patterns of trait-influencing loci in the genome for complex traits.[16] To differentiate SNPs in the six genic categories from SNPs in other annotation categories, a multiple regression was performed with height GWAS summary values (log of z^2 after intergenic inflation control) as the dependent variable. The LD-weighted genic annotation score of each SNP was pre-multiplied by the genotypic

variance (f * (1 - f)) of that SNP (f: minor allele frequency).[8] These scores, from the six genic categories, were the predictors. Thus, the predicted z^2 values ($\hat{z}^2$) represent the degree of enriched polygenic effects that are predicted by the genetic variance of a combination of the six genic categories. The $\hat{z}^2$ values were then used to partition the genome evenly into genic and intergenic regions. SNPs with $\hat{z}^2$ values higher than the median were assigned to the genic category. The results obtained from this type of genomic partitioning were very similar to those obtained with the first method (genic vs. intergenic [± 20kb]) but demonstrated more significant findings. The results are shown in Fig. 2(b), Supplementary Table 5 for the GRM < 0.025 sample and Supplementary Fig. 2(b) and Supplementary Table 8 for the GRM < 0.1 sample.

PING Methods:

Data used in the preparation of this article were obtained from the Pediatric Imaging, Neurocognition and Genetics (PING) Study database (http://ping.chd.ucsd.edu/). PING was launched in 2009 by the National Institute on Drug Abuse (NIDA) and the Eunice Kennedy Shriver National Institute Of Child Health & Human Development (NICHD) as a 2-year project of the American Recovery and Reinvestment Act. The primary goal of PING has been to create a data resource of highly standardized and carefully curated magnetic resonance imaging (MRI) data, comprehensive genotyping data, and developmental and neuropsychological assessments for a large cohort of developing children aged 3 to 20 years. The scientific aim of the project is, by openly sharing these data, to amplify the power and productivity of investigations of healthy and disordered development in children, and to increase understanding of the origins of variation in neurobehavioral phenotypes. For up-to-date information, see http://ping.chd.ucsd.edu/.

ADNI Methods:

Data used in the preparation of this article were obtained from the Alzheimer's Disease Neuroimaging Initiative (ADNI) database (adni.loni.usc.edu). The ADNI was launched in 2003 by the National Institute on Aging (NIA), the National Institute of Biomedical Imaging and Bioengineering (NIBIB), the Food and Drug Administration (FDA), private pharmaceutical companies and non-profit organizations, as a $60 million, 5-year public-private partnership. The primary goal of ADNI has been to test whether serial magnetic resonance imaging (MRI), positron emission tomography (PET), other biological markers, and clinical and neuropsychological assessment can be combined to measure the progression of mild cognitive impairment (MCI) and early Alzheimer's disease (AD). Determination of sensitive and specific markers of very early AD progression is intended to aid researchers and clinicians to develop new treatments and monitor their effectiveness, as well as lessen the time and cost of clinical trials.

The Principal Investigator of this initiative is Michael W. Weiner, MD, VA Medical Center and University of California – San Francisco. ADNI is the result of efforts of many co-investigators from a broad range of academic institutions and private corporations, and subjects have been recruited from over 50 sites across the U.S. and Canada. The initial goal of ADNI was to recruit 800 subjects but ADNI has been followed by ADNI-GO and ADNI-2. To date these three protocols have recruited over 1500 adults, ages 55 to 90, to participate in the research, consisting of cognitively normal older individuals, people with early or late MCI, and people with early AD. The follow up duration of each group is specified in the protocols for ADNI-1, ADNI-2 and

ADNI-GO. Subjects originally recruited for ADNI-1 and ADNI-GO had the option to be followed in ADNI-2. For up-to-date information, see www.adni-info.org.

Supplementary References

1. Espeseth, T. *et al.* Imaging and cognitive genetics: the Norwegian Cognitive NeuroGenetics sample. *Twin Res Hum Genet* **15**, 442-52 (2012).
2. Yang, J. *et al.* Common SNPs explain a large proportion of the heritability for human height. *Nat Genet* **42**, 565-9 (2010).
3. Speed, D., Hemani, G., Johnson, M.R. & Balding, D.J. Improved heritability estimation from genome-wide SNPs. *Am J Hum Genet* **91**, 1011-21 (2012).
4. Lynch, M. & Walsh, B. *Genetics and Analysis of Quantitative Traits*, (Sinauer Associates Inc., 1998).
5. Vinkhuyzen, A.A., Wray, N.R., Yang, J., Goddard, M.E. & Visscher, P.M. Estimation and partition of heritability in human populations using whole-genome analysis methods. *Annu Rev Genet* **47**, 75-95 (2013).
6. Yang, J., Zaitlen, N.A., Goddard, M.E., Visscher, P.M. & Price, A.L. Advantages and pitfalls in the application of mixed-model association methods. *Nat Genet* **46**, 100-6 (2014).
7. Kang, H.M. *et al.* Variance component model to account for sample structure in genome-wide association studies. *Nat Genet* **42**, 348-54 (2010).
8. Schork, A.J. *et al.* All SNPs are not created equal: genome-wide association studies reveal a consistent pattern of enrichment among functionally annotated SNPs. *PLoS Genet* **9**, e1003449 (2013).
9. Lee, J.J. & Chow, C.C. Conditions for the validity of SNP-based heritability estimation. *Hum Genet* **133**, 1011-22 (2014).
10. Visscher, P.M. *et al.* Statistical power to detect genetic (co)variance of complex traits using SNP data in unrelated samples. *PLoS Genet* **10**, e1004269 (2014).
11. Stein, J.L. *et al.* Identification of common variants associated with human hippocampal and intracranial volumes. *Nat Genet* **44**, 552-561 (2012).
12. Kremen, W.S. *et al.* Genes, environment, and time: The Vietnam Era Twin Study of Aging (VETSA). *Twin Research and Human Genetics* **9**, 1009-1022 (2006).
13. Kremen, W.S. *et al.* Genetics of brain structure: contributions from the Vietnam Era Twin Study of Aging. *Am J Med Genet B Neuropsychiatr Genet* **162B**, 751-61 (2013).
14. Eaves, L.J., Last, K.A., Young, P.A. & Martin, N.G. Model-fitting approaches to the analysis of human behavior. *Heredity* **41**, 249-320 (1978).
15. Neale, M.C. & Cardon, L.R. *Methodology for genetic studies of twins and families*, (Kluwer Academic Publishers, Dordrecht, The Netherlands, 1992).
16. Lango Allen, H. *et al.* Hundreds of variants clustered in genomic loci and biological pathways affect human height. *Nature* **467**, 832-8 (2010).
